# Osteosarcopenia in metabolic dysfunction–associated steatotic liver disease: from mechanisms to management

**DOI:** 10.3389/fendo.2025.1706068

**Published:** 2025-12-11

**Authors:** Aili Fan, Jie Zhang, Qing Ye

**Affiliations:** 1 Department of Gastroenterology/Hepatology, Tianjin University Central Hospital/The Third Central Hospital Affiliated with Nankai University, Tianjin, China; 2 School of Medicine, Nankai University, Tianjin, China; 3 Tianjin Key Laboratory of Extracorporeal Life Support for Critical Diseases, Tianjin Institute of Hepatobiliary Disease, Tianjin, China

**Keywords:** osteosarcopenia, osteoporosis, sarcopenia, metabolic dysfunction – associated steatotic liver disease, mechanism, management

## Abstract

Osteosarcopenia, the coexistence of osteoporosis and sarcopenia, is an emerging and underrecognized complication in patients with metabolic dysfunction-associated steatotic liver disease (MASLD). While muscle and bone loss have been individually observed in MASLD, their combined impact remains poorly addressed in clinical practice. This review outlines the epidemiology, pathophysiological mechanisms, clinical relevance, and current strategies for diagnosing and managing osteosarcopenia in MASLD. Shared pathogenic pathways, including insulin resistance, chronic inflammation, hormonal imbalance, and gut dysbiosis, create a vicious cycle contributing to musculoskeletal degradation and liver disease progression. We highlight the need for proactive screening of osteosarcopenia, and using standardized assessment tools. A multidimensional therapeutic approach, encompassing nutrition, exercise, pharmacotherapy, and emerging metabolic and gut-targeted interventions, may improve not only musculoskeletal health but also hepatic and systemic outcomes. Future studies are warranted to improve long-term prognosis for both osteosarcopenia and MASLD.

## 1 Introduction

Metabolic dysfunction–associated steatotic liver disease (MASLD), formerly known as non-alcoholic fatty liver disease (NAFLD), is a major chronic liver disease characterized by hepatocellular dysregulation of lipid and glucose metabolism in the context of cardiometabolic dysfunction encompassing insulin resistance, atherogenic dyslipidemia, dysglycemia, hypertension. It affects about 38% of adults globally and is especially common in non-geriatric individuals with overweight or obesity. The condition evolves along a biological continuum from isolated steatosis to metabolic dysfunction–associated steatohepatitis (MASH), progressive fibrosis and cirrhosis, with a subset advancing to hepatocellular carcinoma. Beyond the liver, MASLD imposes a multisystem burden, accompanied by endocrine and immunometabolic disturbances that worsen glycemic control and accelerate atherosclerotic cardiovascular disease. Meanwhile, patients with MASLD also frequently exhibit sarcopenia and low bone mass, driven by chronic inflammation, insulin resistance and endocrine dysregulation (1, 2).

Depending on the population studied and the diagnostic criteria applied, approximately 12–43% of individuals with MASLD meet sarcopenia criteria, compared with ~8–10% in metabolically healthy controls (3, 4). The prevalence appears even higher, ranging from 30% to 35%, in patients with non-alcoholic steatohepatitis (NASH), the inflammatory and progressive form of MASLD (3, 5). In parallel, a meta-analysis involving over 30,000 individuals found that MASLD was associated with a ~17% increase in the odds of low bone mineral density (BMD) or osteoporosis, along with an elevated fracture risk (6). Together, these findings emphasize that musculoskeletal deficits cluster in MASLD and support integrated assessment of musculoskeletal health alongside liver-centered care.

Osteosarcopenia, is a recently proposed geriatric syndrome characterized by the co-occurrence of osteoporosis and sarcopenia in a single individual (7, 8). In the context of MASLD, insulin resistance sustains hyperinsulinemia and impairs anabolic signaling. Chronic low-grade inflammation and disturbances in the growth hormone–IGF-1, sex-steroid and vitamin-D axes reduce muscle protein synthesis and bone remodeling. Although osteoporosis and sarcopenia each contribute to frailty and disability, their coexistence appears synergistic and is associated with higher risks of falls, fractures, loss of independence and mortality than either condition alone (9, 10,) (11–14) This pathophysiological and clinical overlap suggests that osteosarcopenia may represent a particularly relevant musculoskeletal phenotype in MASLD.

Despite mounting evidence, osteosarcopenia remains under-recognized in routine practice. A lack of standardized screening criteria, limited clinician awareness, and the absence of unified treatment protocols all contribute to missed opportunities for early intervention (15). Evidence on MASLD-associated osteosarcopenia is also fragmented. Here, in this Review, we examine the evolving understanding of osteosarcopenia in the context of MASLD, with a focus on its underlying mechanisms, clinical significance, diagnostic approaches, and emerging management strategies.

## 2 The significance of osteosarcopenia in MASLD

### 2.1 Sarcopenia in MASLD

Sarcopenia is now recognized as a progressive and generalized skeletal muscle disorder characterized by the loss of muscle strength and muscle mass, leading to impaired physical performance and increased risks of falls, fractures, disability and mortality (16). Epidemiologically, the overall prevalence of sarcopenia is estimated at roughly 10–27% worldwide in general population cohorts of adults ≥60 years and at lower but non-negligible rates in younger adults (15, 17), with country-specific data suggesting values around 9.9% in Japan, 13.1% in Korea, 17.0% in Brazil and about 20.7% in China, which demonstrates important geographical and ethnic differences (18).

On a biological level, sarcopenia develops when the capacity of skeletal muscle to repair and maintain itself can no longer keep pace with ongoing loss. With advancing age and reduced physical activity, muscles gradually become less responsive to growth stimuli, while chronic low-grade inflammation and hormonal changes impair regeneration and favor muscle wasting. In metabolic conditions such as obesity, type 2 diabetes and MASLD, insulin resistance and fat accumulation within muscle further aggravate this imbalance, so that loss of muscle mass and strength occurs earlier and progresses more rapidly than in healthy ageing.

In individuals with MASLD, sarcopenia is substantially more common than in the general population (19, 20). A meta-analysis of 19 studies confirmed a significant association between MASLD and sarcopenia, with a pooled odds ratio (OR) of ~1.33 for MASLD overall and ~2.4 for NASH specifically (19, 21, 22). Longitudinal cohort studies suggest that low skeletal muscle mass may not only result from MASLD, but may also precede and predict its development. In two large prospective cohorts with 7–10 years of follow-up, baseline skeletal muscle index was inversely associated with incident MASLD, while higher muscle mass predicted spontaneous resolution of hepatic steatosis (23). Another mendelian randomization analyses further support a potential causal link between reduced lean mass and increased MASLD risk (22). Although a few studies have reported non-significant associations—likely reflecting differences in sarcopenia definitions or confounding factors—the overall body of evidence consistently implicates low muscle mass as both a risk factor and consequence of MASLD (24). Taken together, these data indicate a bidirectional relationship between sarcopenia and MASLD.

Of particular concern is sarcopenic obesity, a phenotype defined by the coexistence of obesity and sarcopenia, that is, excess adiposity together with reduced muscle strength and/or impaired physical performance and relatively low muscle mass (25, 26). In individuals with MASLD, sarcopenic obesity is especially relevant because obesity, ectopic fat deposition and insulin resistance frequently coincide with subtle but clinically important impairments in muscle function. From a clinical standpoint, however, sarcopenic obesity is difficult to recognize: absolute lean mass may remain within the “normal” range, excess body weight can mask relative muscle depletion, and routine assessments based on BMI or waist circumference alone do not capture muscle quality or strength (27, 28). These diagnostic challenges are compounded in high-risk subgroups such as individuals with type 2 diabetes, in whom sarcopenia, visceral adiposity and MASLD frequently cluster. Consequently, a substantial proportion of patients with MASLD and sarcopenic obesity are likely to be underdiagnosed unless targeted evaluation of muscle strength and body composition is incorporated into clinical practice. This under-recognition has important implications, because sarcopenia—particularly in its sarcopenic obesity form—represents one of the two core components of osteosarcopenia in MASLD.

### 2.2 Low bone mass and osteoporosis in MASLD

Osteoporosis is classically defined by the WHO as a bone mineral density (BMD) 2.5 standard deviations or more below the young adult mean at the lumbar spine or hip (T-score ≤ –2.5), whereas low bone mass (osteopenia) refers to a T-score between –1.0 and –2.5. Globally, osteoporosis affects almost one in five adults, with a pooled prevalence of around 18% in the general population, and a higher burden in women (23%) than in men (12%), with considerable variation across regions and ethnic groups. Against this background, MASLD has emerged as an additional and potentially modifiable determinant of skeletal fragility (29).

Mechanistically, MASLD and osteoporosis share several pathophysiological pathways. Chronic low-grade inflammation, oxidative stress and insulin resistance, which are key drivers of MASLD, promote osteoclast activation and inhibit osteoblast function, leading to an imbalance in bone remodelling (6, 30–32). Ectopic fat accumulation within the bone marrow may further favor adipogenesis over osteoblast differentiation, while alterations in adipokines and myokines link disturbances in liver, adipose tissue and muscle to bone loss. In more advanced stages of liver disease, particularly NASH with significant fibrosis or cirrhosis, reduced hepatic synthesis of IGF-1, impaired activation of vitamin D, hypogonadism, malnutrition and cholestasis further compromise bone formation and mineralization, contributing to the classical picture of hepatic osteodystrophy (6, 30–32).

Epidemiological observations across the life course broadly support these mechanistic links. In children and adolescents, MASLD—particularly when accompanied by NASH—has been linked to significantly lower bone density Z-scores compared with healthy peers, suggesting that liver disease may interfere with peak bone accrual during growth (6, 33) In adults, MASLD has been associated with decreased BMD at specific skeletal sites, including reductions in calcaneal bone strength in older women and non-obese individuals (34). In postmenopausal women, the severity of liver fibrosis correlates positively with osteoporosis risk, potentially reflecting synergistic effects of estrogen deficiency and liver-derived pro-inflammatory mediators (30, 35, 36). n high-risk metabolic groups, such as individuals with type 2 diabetes, MASLD is highly prevalent and appears to confer additional bone fragility on top of the already increased fracture risk associated with diabetes, although data remain limited and partly conflicting.

However, not all studies have found a clear association. In some cases, the link between MASLD and bone loss appears attenuated after adjustment for body mass index or visceral adiposity, underscoring the role of fat distribution as a confounding factor (37). Despite this heterogeneity, most biopsy-based and elastography studies report that low BMD, osteoporosis and vertebral deformities are more frequent in patients with histologically proven NASH or significant fibrosis (F ≥ 3) than in those with simple steatosis, and that fracture rates are highest in individuals with advanced fibrosis and cirrhosis (33). In these stages, MASLD frequently coexists with sarcopenia, hypogonadism, vitamin D deficiency and poor nutritional status, further amplifying skeletal fragility and contributing to an osteosarcopenic phenotype. Taken together, current evidence supports MASLD—particularly when inflammatory activity and fibrosis are present—as an important determinant of impaired skeletal health, while highlighting the need for well-designed longitudinal and mechanistic studies to disentangle the independent contributions of steatosis, inflammation and fibrosis to bone metabolism and fracture risk. n high-risk metabolic groups, such as individuals with type 2 diabetes, MASLD is highly prevalent and appears to confer additional bone fragility on top of the already increased fracture risk associated with diabetes, although data remain limited and partly conflicting.

### 2.3 Osteosarcopenia in MASLD

With the rapid aging of the global population, osteosarcopenia is gaining recognition as a clinically important yet underdiagnosed entity, defined by the coexistence of low bone mass and low muscle mass and/or strength (38). The prevalence of osteosarcopenia increases steeply with age (38). For instance, in Iran, the prevalence in men escalates from approximately 14% at ages 60–64 to nearly 59% at age 75 and above; among women, the corresponding increase is from 20% to 48% (39, 40). Community-based studies across different geographic regions reveal marked variation in prevalence — approximately 4.7% in Japan, 7–13% in China, 28% in Germany, and up to 40% in Australia (41–45). Despite this heterogeneity, these data consistently show that osteosarcopenia is relatively common among older adults and becomes increasingly prevalent with advancing age (45–47), which underscores the need for greater clinical awareness, standardized diagnostic approaches, and targeted interventions to mitigate its health burden.

In older adults recovering from hip fractures, osteosarcopenia is both common and consequential (48, 49) with a prevalence of approximately 27% in this group, and associated with a substantially elevated one-year mortality (~15%) compared with that observed in patients with either osteoporosis or sarcopenia alone (~5–9%) (14, 40, 48). Moreover, individuals with osteosarcopenia also exhibit more severe impairments in mobility and physical performance, higher rates of frailty and more pronounced depressive symptoms, highlighting the multidimensional burden of this condition (50).

Although direct data on osteosarcopenia in MASLD remain scarce, converging evidence from the sarcopenia and osteoporosis literature suggests that this combined phenotype may be relatively common (51). Given the elevated prevalence of each condition in MASLD, particularly among older adults with long-standing metabolic dysfunction, it is plausible that osteosarcopenia is underrecognized yet clinically relevant in this population (52). Supporting this notion, one analysis found that men in the highest tertile of fatty liver index exhibited significantly lower BMD at multiple sites and had a ~2.5-fold higher risk of low bone mass or osteoporosis, even after adjusting for insulin resistance (53, 54). Although such studies did not specifically define “osteosarcopenia,” they underscore the overlap in metabolic risk factors and pathophysiological mechanisms shared between sarcopenia and osteoporosis in MASLD.

As MASLD progresses to advanced stages such as significant fibrosis, cirrhosis, or hepatocellular carcinoma, the risk of osteosarcopenia increases substantially (51, 55, 56). Sarcopenia affects approximately one-third of patients with cirrhosis overall, with prevalence rising sharply with disease severity—up to 45–90% in decompensated cases—and particularly high rates observed in NASH-related cirrhosis (57). Osteoporosis is also common, affecting 20–50% of patients with advanced liver disease, and is linked to increased fracture risk, especially in those with metabolic or cholestatic etiologies (57–59). These musculoskeletal complications result from various mechanisms, including chronic inflammation, malnutrition, hypogonadism, reduced IGF-1 synthesis, and immobility (60, 61).

Emerging data now highlight the combined burden of sarcopenia and osteoporosis—osteosarcopenia—as a distinct and high-risk phenotype in cirrhosis (56). In mixed-etiology liver disease cohorts, osteosarcopenia has been reported in ~17–22% of patients, and is closely associated with vertebral fractures and clinical frailty (62, 63). Among frail individuals with chronic liver disease, nearly half may have osteosarcopenia (64). Although dedicated studies in MASLD-related cirrhosis remain limited, the convergence of overlapping metabolic risk factors and shared pathophysiology strongly suggests that osteosarcopenia is prevalent yet underrecognized in this population (62). Thus, characterizing its clinical course and prognostic implications in MASLD represents a key area for future research.

## 3 Pathophysiological mechanisms linking MASLD, sarcopenia, and osteoporosis

**Figure 1:**
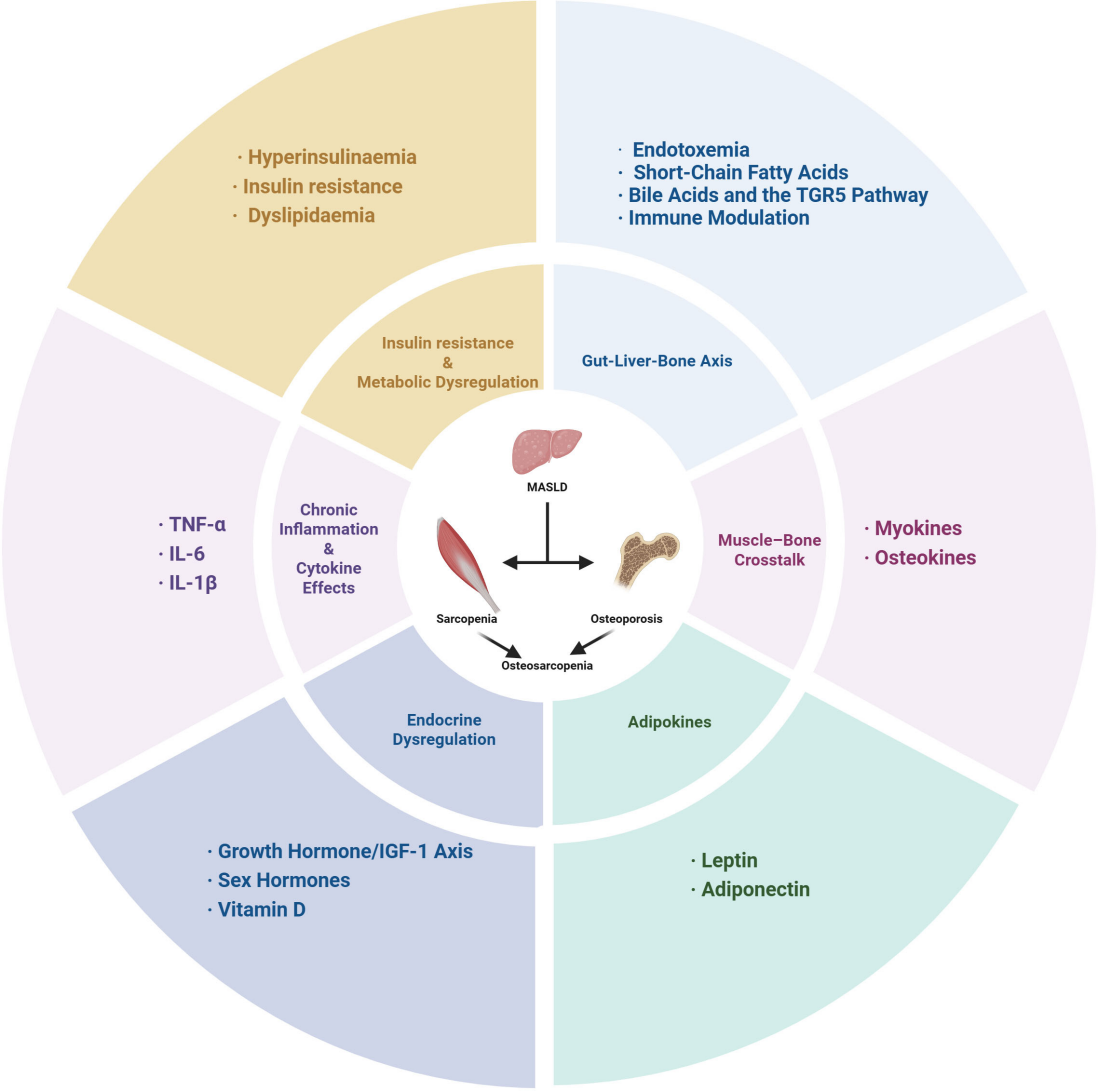
Pathophysiological mechanisms linking MASLD to osteosarcopenia.

The metabolic and endocrine disturbances caused by MASLD can drive both muscle wasting and bone loss through multiple interrelated mechanisms, which involve not only the systemic effects of hepatic steatosis itself but also contributions from coexisting obesity, chronic inflammation, insulin resistance, and age-related endocrine dysregulation (**Figure 1**).

### 3.1 Insulin resistance and metabolic dysregulation

MASLD, as the hepatic manifestation of metabolic syndrome, is intrinsically linked to systemic insulin resistance (IR), which exerts detrimental effects on both muscle and bone homeostasis (65, 66). In skeletal muscle, IR impairs glucose uptake and disrupts protein metabolism by suppressing anabolic signaling and promoting proteolysis, thereby contributing to muscle atrophy and reduced strength. Simultaneously, chronic hyperinsulinemia and hyperglycemia, common features in MASLD, negatively impact bone metabolism (34), where these metabolic insults impair insulin/IGF-1 signaling to osteoblasts, thereby inhibiting bone formation, and favor adipogenic differentiation within the bone marrow niche at the expense of osteogenesis (67, 68).

Clinical evidence supports this mechanistic link: MASLD patients with type 2 diabetes—representing a state of pronounced insulin resistance—tend to exhibit lower muscle mass and strength, along with a higher risk of osteoporosis, compared with non-diabetic counterparts (34, 69). Collectively, the metabolic milieu of MASLD—characterized by hyperinsulinemia, hyperglycemia, and dyslipidemia—is deleterious to both myocytes and osteoblasts (70). Therefore, while insulin resistance often coexists with chronic inflammation and other metabolic disturbances, it nonetheless forms a central pathophysiological axis in the development of osteosarcopenia in MASLD (71).

### 3.2 Chronic inflammation and cytokine effects

MASLD is frequently accompanied by chronic, low-grade systemic inflammation, which plays a central role in mediating muscle and bone catabolism (72, 73). Hepatic steatosis and excess visceral adiposity promote the release of pro-inflammatory cytokines—most notably tumor necrosis factor-alpha (TNF-α), interleukin-6 (IL-6), and interleukin-1β (IL-1β)—which exert deleterious effects on musculoskeletal tissues (72–76).

In skeletal muscle, these cytokines activate catabolic pathways such as NF-κB signaling, enhance ubiquitin–proteasome-mediated protein degradation (77), and promote fiber atrophy, all of which contribute to the development of chronic disease–associated sarcopenia (78). In bone, inflammatory mediators disrupt the delicate balance of bone remodeling (79). TNF-α impairs osteoblast differentiation and survival while upregulating RANKL expression, thereby promoting osteoclastogenesis (80). Similarly, IL-6 and IL-1β stimulate osteoclast activity and bone resorption, accelerating bone loss (76, 81).

Both experimental models of MASLD and clinical observations strongly support inflammation as a mechanistic link connecting liver dysfunction with musculoskeletal degeneration (79). Notably, anti-inflammatory therapies—such as TNF-α or IL-6 inhibitors—have been hypothesized to improve both hepatic and skeletal outcomes in this context, although definitive clinical trial evidence is still lacking (82). Nonetheless, the conceptual framework is compelling: dampening systemic inflammation may offer dual benefits for both the liver and the musculoskeletal system (83).

In summary, persistent inflammation in MASLD serves as a common mechanistic driver of both muscle proteolysis and bone resorption, thus forming a key pathophysiological bridge between steatotic liver injury and osteosarcopenia.

### 3.3 Endocrine dysregulation

#### 3.3.1 Growth hormone/IGF-1 axis

The GH/IGF-1 axis plays a central anabolic role in musculoskeletal health. IGF-1, primarily synthesized by the liver in response to GH, promotes both muscle protein synthesis and bone formation (84). In MASLD, particularly in the presence of advanced fibrosis, circulating IGF-1 levels are frequently reduced due to impaired hepatic production (84). Animal models of MASLD have demonstrated concomitant muscle atrophy and low IGF-1 levels, directly linking hepatic steatosis to muscle wasting (21). In humans, IGF-1 levels decline with cirrhosis progression and correlate with increased frailty (85). Restoration of liver function—such as after transplantation—can normalize the GH/IGF-1 axis and improve muscle mass (86). Furthermore, a small randomized trial in hypogonadal men with cirrhosis found that testosterone therapy, which can enhance IGF-1, significantly increased lean mass and improved BMD after 12 months (86). These findings suggest that restoring GH/IGF-1 signaling may benefit both muscle and bone, although the potential to reverse long-standing sarcopenia remains uncertain.

#### 3.3.2 Sex hormones

Hypogonadism is common in chronic liver disease and contributes to musculoskeletal decline (87). Men with MASLD or cirrhosis often exhibit low testosterone levels, which are associated with reduced muscle mass, strength, and BMD (88). In postmenopausal women, the absence of estrogen removes its bone-protective effects, accelerating bone loss. Observational studies have shown that in postmenopausal women with MASLD, higher fibrosis stage is associated with increased osteoporosis risk—likely reflecting the combined impact of estrogen deficiency and hepatic inflammation (87).

Testosterone replacement in male cirrhosis patients has been associated not only with improvements in muscle and bone mass, but also with fewer hepatic decompensation events and improved one-year survival (77). These findings suggest that correcting sex hormone deficiencies may offer systemic benefits. However, hormone therapy must be considered with caution—monitoring for potential prostate-related adverse effects in men and thromboembolic risk in women (89). Selective estrogen receptor modulators (e.g., raloxifene) have been shown to improve BMD in postmenopausal women and may be considered when osteoporosis coexists, although their impact on liver disease remains unclear (89). Overall, sex hormone deficiency is an important contributor to osteosarcopenia in MASLD (89).

#### 3.3.3 Vitamin D

The liver is a key organ in vitamin D metabolism, catalyzing 25-hydroxylation of cholecalciferol (90). Vitamin D deficiency is highly prevalent in MASLD and is independently associated with greater steatosis, fibrosis, and systemic inflammation (91). As a regulator of calcium homeostasis and bone turnover, insufficient 25(OH)D impairs calcium absorption, reduces osteoblast mineralization, and increases fracture risk (91).

Experimental studies suggest that vitamin D deficiency also exacerbates hepatic steatosis and inflammation (92). Clinically, correction of vitamin D deficiency is a standard component of MASLD management—particularly in patients at risk of bone loss (92). Targeting serum 25(OH)D levels >30 ng/mL may improve BMD and modestly benefit muscle function, as skeletal muscle expresses vitamin D receptors and severe deficiency can cause proximal muscle weakness (93). Although clinical trials on vitamin D supplementation in MASLD have yielded mixed results, some have reported improvements in liver enzymes and hepatic steatosis (93). Given its safety and skeletal benefits, vitamin D repletion is generally recommended when deficiency is identified.

In summary, MASLD creates a hormonally adverse internal milieu—marked by low IGF-1, sex hormone deficiency, and inadequate vitamin D—that collectively contributes to the development of osteosarcopenia. Although causality remains complex and multifactorial, addressing endocrine disturbances may represent a rational component in the broader management of musculoskeletal decline in MASLD.

### 3.4 Adipokines in MASLD

Adipose tissue, far from being inert, functions as a dynamic endocrine organ (94). In the setting of obesity and MASLD, adipocyte expansion alters the secretion of adipokines—bioactive molecules that modulate liver function, systemic metabolism, and, notably, muscle and bone homeostasis (95). Among these, leptin and adiponectin have received the most attention due to their contrasting metabolic profiles.

#### 3.4.1 Leptin

Leptin levels are elevated in obesity and MASLD, and its actions appear context-dependent (96). In the liver, leptin exerts pro-inflammatory and pro-fibrotic effects, promoting hepatic stellate cell activation and fibrosis. Its impact on bone is complex: peripherally, leptin may stimulate osteogenesis, but centrally—via hypothalamic signaling—it suppresses bone formation (97, 98). These opposing effects contribute to conflicting clinical observations, with some studies paradoxically linking high leptin levels to reduced fracture risk. This may reflect skeletal loading from higher fat mass, rather than a direct protective effect (99).

Leptin’s role in muscle metabolism remains less clearly defined. Chronically elevated leptin may induce leptin resistance and sustain a pro-inflammatory environment, potentially exacerbating muscle catabolism. However, direct evidence of its deleterious effects on muscle remains limited. Overall, whether hyperleptinemia in MASLD is protective or harmful to musculoskeletal health remains unresolved—it may exert mixed and tissue-specific effects (100). Nonetheless, elevated leptin is a hallmark of metabolic stress, and in patients with osteosarcopenia and MASLD, its role warrants further investigation.

#### 3.4.2 Adiponectin

In contrast, adiponectin—typically reduced in central obesity and MASLD—is considered metabolically protective (95), which enhances insulin sensitivity and exerts anti-inflammatory effects through activation of AMPK and PPAR-α signaling pathways in hepatocytes, reducing steatosis and hepatic inflammation (101). In bone, experimental studies suggest adiponectin supports osteoblast differentiation and inhibits osteoclastogenesis (102). In ovariectomized rat models, adiponectin administration prevented both bone loss and muscle atrophy, hinting at a potential therapeutic role (101).

Paradoxically, however, human epidemiological studies often associate higher circulating adiponectin levels with lower bone density and increased fracture risk—a phenomenon sometimes referred to as the “adiponectin paradox” (101). This may reflect compensatory adiponectin elevation in frail individuals, rather than a causal role in bone loss. While adiponectin is generally considered beneficial for hepatic and metabolic health, its relationship with muscle and bone in osteosarcopenia remains complex (99). Strategies to enhance adiponectin signaling, such as receptor agonists, have been proposed, but remain experimental.

### 3.5 Muscle–bone crosstalk

Beyond adipose-derived signals, muscle and bone directly communicate through molecular mediators—myokines and osteokines—that influence each other’s function (103). For instance, irisin, a myokine released during exercise, has been shown *in vitro* to stimulate osteoclast activity, yet higher circulating irisin in humans correlates with increased BMD, suggesting a broader role in supporting bone formation or delaying senescence (104). Conversely, osteocalcin—a hormone secreted by osteoblasts—has demonstrated capacity to enhance muscle regeneration and improve systemic insulin sensitivity, linking bone function to broader metabolic health (105). Such muscle–bone signaling pathways likely contribute to the pathophysiology of osteosarcopenia in MASLD, although their specific roles remain underexplored (106, 107). However, preliminary evidence suggests that interventions targeting one tissue can confer benefits to the other (108, 109). For example, resistance exercise improves both muscle mass and BMD, while increased muscle mass has been associated with slower progression of MASLD (51).

### 3.6 Gut-liver-bone axis

The gut microbiota plays a central role in regulating host metabolism and immunity. Microbial dysbiosis has been implicated in both the progression of MASLD and in bone loss, suggesting a pathophysiological connection known as the “gut–liver axis.” (79).

#### 3.6.1 Endotoxemia

Overgrowth of Gram-negative bacteria and increased intestinal permeability (“leaky gut”) can facilitate the translocation of lipopolysaccharide (LPS) into the portal circulation (110). LPS triggers hepatic inflammation and elevates systemic pro-inflammatory cytokines such as IL-1β and TNF-α. These cytokines upregulate RANKL expression and promote osteoclast activation, leading to increased bone resorption (111). This establishes a mechanistic link between impaired gut barrier function and MASLD-related bone loss. Animal studies further demonstrate that chronic low-dose LPS exposure or gut-derived inflammation can induce osteoporosis-like phenotypes (112). Thus, in MASLD, persistent exposure to gut-derived endotoxins may accelerate skeletal deterioration (112, 113).

#### 3.6.2 Short-chain fatty acids

Gut microbes ferment dietary fiber to produce SCFAs—primarily acetate, propionate, and butyrate—which have widespread systemic effects (114). Butyrate enhances intestinal barrier integrity and exhibits anti-inflammatory effects, including increased GLP-1 secretion, which is beneficial in MASLD (114). In contrast, excess acetate and propionate may promote inflammation in certain contexts (114). SCFAs also act via endocrine pathways to modulate bone metabolism: they stimulate hepatic IGF-1 production and support an osteogenic immune milieu, for instance, by expanding regulatory T cells (Tregs) (115). Variations in microbiome composition—and thus SCFA profiles—may help explain interindividual differences in MASLD-related bone loss (115). Enhancing butyrate-producing microbes through prebiotic fiber intake is a promising dual-targeted approach to support both liver and bone health (116). Conversely, overrepresentation of microbes producing acetate/propionate may worsen metabolic inflammation. Altogether, SCFAs serve as key signaling molecules bridging the gut with the liver and skeleton.

#### 3.6.3 Bile acids and the TGR5 pathway

The gut microbiota transforms primary bile acids into secondary bile acids, modulating the bile acid pool and activating receptors such as FXR and TGR5. TGR5 activation—e.g., by certain secondary bile acids like ursodeoxycholic acid derivatives—stimulates GLP-1 secretion from intestinal L-cells and calcitonin release from thyroid C-cells (116). GLP-1 improves insulin sensitivity and metabolic homeostasis, while calcitonin directly inhibits osteoclast activity, thereby reducing bone resorption (117). In MASLD, dysbiosis may reduce the abundance of microbes that generate beneficial secondary bile acids, leading to lower levels of GLP-1 and calcitonin and weakened bone-protective signals (116). Preliminary studies support this concept, spurring interest in gut-targeted therapies—such as probiotics, prebiotics, or bile acid analogs—that could simultaneously address hepatic and skeletal dysfunction (116). Although clinical evidence remains limited, modulating the intestinal bile acid–TGR5–GLP-1/calcitonin axis represents a novel and attractive therapeutic strategy.

#### 3.6.4 Immune modulation

Microbial dysbiosis also alters systemic immune balance. Disruption of the gut microbiota can reduce bone-protective Tregs and increase Th17 cells, which promote osteoclastogenesis—an immune imbalance observed in autoimmune and inflammatory conditions like rheumatoid arthritis and likely relevant to MASLD-related bone loss (118). Elevated circulating levels of TNF-α and IL-17, often seen in MASLD, further exacerbate bone resorption (119). Additionally, certain gut bacteria synthesize vitamins such as K2, essential for osteocalcin carboxylation and bone mineralization. Loss of these bacteria may lead to vitamin K deficiency and impaired skeletal integrity (119). While these immune-related mechanisms are well documented in other diseases, direct evidence in MASLD-associated osteosarcopenia is sparse (119). Nonetheless, a pro-inflammatory, bone-resorptive immune profile driven by dysbiosis is a plausible contributor to accelerated osteopenia in MASLD and warrants further investigation.

In summary, MASLD induces a constellation of systemic disturbances—including insulin resistance, chronic inflammation, hormonal imbalances, and gut-derived signals—that collectively create a catabolic environment favoring muscle wasting and bone loss (119). Some mechanisms, such as inflammation and vitamin D deficiency, are well-established, while others—like microbiome-derived metabolites and gut–bone immune cross-talk—represent emerging and promising areas of research. A deeper understanding of the gut–liver–bone axis may ultimately enable targeted interventions that not only address hepatic pathology but also mitigate musculoskeletal complications such as osteosarcopenia.

## 4 Diagnosis of osteosarcopenia in MASLD

Early recognition and accurate diagnosis of muscle and bone loss in patients with MASLD are essential for timely intervention and prevention of adverse outcomes (54). This section outlines the diagnostic approach to sarcopenia, osteoporosis, and their overlap—osteosarcopenia—in the MASLD population.

### 4.1 Sarcopenia assessment in MASLD

Sarcopenia is now defined, according to the revised European Working Group on Sarcopenia in Older People (EWGSOP2), as a progressive and generalized skeletal muscle disorder characterized by low muscle strength and low muscle quantity or quality, with impaired physical performance indicating severe disease. Low handgrip strength is considered the primary indicator of “probable” sarcopenia, which is then confirmed by documentation of low muscle mass; common cut-offs include handgrip strength <27 kg in men and <16 kg in women and appendicular lean mass/height² <7.0 kg/m² in men and <5.5 kg/m² in women, with Asian Working Group for Sarcopenia (AWGS) thresholds providing ethnicity-specific cut-offs for Asian populations. Muscle function is further characterized by physical performance tests such as gait speed (e.g., ≤0.8–1.0 m/s), chair stand tests, or Short Physical Performance Battery scores. Body composition can be assessed by dual-energy X-ray absorptiometry (DXA) or bioelectrical impedance analysis (BIA) to estimate appendicular lean mass, and by cross-sectional imaging (CT or MRI) at the third lumbar vertebra (L3) to derive skeletal muscle index (SMI) (120).

In MASLD, the reported prevalence of sarcopenia varies widely according to the diagnostic criteria and methods used, but several consistent observations have emerged (121). Relative muscle metrics, such as muscle mass adjusted for body weight or BMI, are more sensitive indicators in this population than absolute muscle mass. For instance, one study showed that muscle mass relative to body weight was significantly associated with MASLD, whereas height-adjusted muscle mass was not, the reported prevalence of sarcopenia varies widely according to the diagnostic criteria and methods used, but several consistent observations have emerged (121). In everyday practice, patients with MASLD may therefore appear to have “normal” muscle quantity when judged by absolute values, yet their muscle mass is functionally inadequate for their body size. CT-based techniques can also detect myosteatosis, defined as fat infiltration within skeletal muscle, which is increasingly recognized as an independent predictor of adverse clinical outcomes in MASLD and chronic liver disease (122).

The concept of sarcopenic obesity is particularly relevant in MASLD, where obesity is highly prevalent and excess adiposity can mask muscle deficiency. Sarcopenic obesity is defined by the coexistence of obesity and sarcopenia – that is, increased fat mass together with low muscle mass and function. A 2022 ESPEN/EASO consensus and a subsequent Nature Reviews Endocrinology overview proposed a two-step approach for sarcopenic obesity: (i) case-finding in individuals with elevated BMI or waist circumference who have risk factors such as advanced age, diabetes, chronic liver disease, or physical frailty; and (ii) confirmation based on objective evidence of low muscle strength plus an adverse body composition phenotype (high adiposity and reduced relative muscle mass). In obese individuals, absolute muscle mass or height-adjusted indices may remain in the “normal” range despite clinically relevant functional impairment; indices that relate muscle mass to body weight or BMI (e.g., appendicular lean mass/weight or appendicular lean mass/BMI) are therefore preferred for defining low muscle quantity in sarcopenic obesity and have been emphasized by recent consensus statements. At present, no dedicated sarcopenia cut-offs have been validated specifically for obese MASLD populations; therefore, current practice applies EWGSOP2/AWGS thresholds for low muscle strength and mass, while preferentially using weight- or BMI-adjusted muscle indices when obesity is present.

From a clinical standpoint, routine screening for sarcopenia is recommended in MASLD, particularly in older adults and those with type 2 diabetes (122). Simple tools like the SARC-F questionnaire or grip strength testing can be used during clinic visits. If screening is positive or clinical suspicion is high (e.g., patient presents with weakness or frailty), formal evaluation with DXA or CT imaging and functional testing should follow. Despite the recommendations of guidelines such as EWGSOP2, sarcopenia screening is still underutilized in hepatology clinics (122). Improving detection is an important step in delivering comprehensive care to MASLD patients.

### 4.2 Bone density evaluation in MASLD

BMD is typically assessed using dual-energy X-ray absorptiometry (DXA), with measurements taken at the lumbar spine and hip (either total hip or femoral neck). According to World Health Organization (WHO) criteria, osteoporosis is defined as a BMD T-score of ≤ –2.5, while osteopenia is defined as a T-score between –1.0 and –2.5 (123). These thresholds also apply to MASLD patients, but the presence of additional risk factors—including vitamin D deficiency, hypogonadism, and chronic inflammation—justifies earlier BMD evaluation than in the general population (123).

Current liver society guidelines recommend BMD screening for all patients with cirrhosis; it is reasonable to extend this to MASLD patients with significant fibrosis, long-standing diabetes, or other risk factors (124). Fracture risk can also be estimated using tools such as FRAX. However, in patients with liver disease FRAX may underestimate risk because it does not explicitly account for frailty or fall risk (125). The FRAX-Plus calculator and the NOGG tool, when used alongside FRAX, partially address these limitations by allowing adjustment for standardized country-specific fall risk and by recognizing severe liver disease as a cause of secondary osteoporosis (126).

If osteoporosis or significant osteopenia is diagnosed, appropriate management—including calcium and vitamin D supplementation, lifestyle interventions, and pharmacotherapy—should be initiated (125). Although it remains unclear whether osteoporosis treatment alters liver disease outcomes, fracture prevention can substantially improve patient quality of life. Additionally, interventions such as resistance training or correcting vitamin D deficiency may benefit both bone and liver health.

### 4.3 Identifying osteosarcopenia: the overlap of sarcopenia and osteoporosis

Osteosarcopenia refers to the co-occurrence of sarcopenia and osteoporosis (127). Although it currently lacks a unified diagnostic definition, it is typically identified by meeting established criteria for both conditions—such as EWGSOP2 for sarcopenia and WHO T-score thresholds for osteoporosis (127).

In clinical practice, recognizing osteosarcopenia requires attention to overlapping risk factors. An older MASLD patient who appears thin or frail, or who has muscle weakness and concurrent risk factors for bone loss (e.g., corticosteroid use, postmenopausal status), is likely to have both sarcopenia and osteoporosis. A practical diagnostic approach is “identify one, screen for the other.” (127) For example, if sarcopenia is diagnosed (e.g., via low grip strength and reduced muscle mass on BIA or CT), perform DXA to assess bone status, while if osteoporosis is identified, assess muscle function through grip strength, gait speed, or muscle mass measurement. This integrated assessment model is well-established in geriatrics and endocrinology but remains underutilized in hepatology (128). Yet, literature increasingly supports considering osteosarcopenia as a distinct high-risk phenotype, associated with greater frailty, falls, fractures, hospitalizations, and mortality compared to either condition alone (127).

While more evidence is needed to determine whether combined treatment improves outcomes, recognizing osteosarcopenia is a crucial first step. For MASLD patients with multimorbidity, especially those who appear frail or functionally impaired, a presumptive diagnosis of osteosarcopenia may help galvanize a more comprehensive and multidisciplinary intervention strategy targeting both muscle and bone health (127).

## 5 Emerging strategies for management and treatment

Effective management of osteosarcopenia in MASLD requires a comprehensive, multimodal approach that simultaneously addresses hepatic steatosis, muscle loss, and bone deterioration. Current strategies draw upon established interventions for MASLD, sarcopenia, and osteoporosis, with a growing emphasis on integrated care (129). This section highlights lifestyle, nutritional, pharmacological, and emerging therapeutic strategies, outlining both evidence-based recommendations and areas of ongoing investigation.

### 5.1 Lifestyle interventions

#### 5.1.1 Weight loss

For individuals with MASLD who are overweight or obese, weight loss is a cornerstone intervention with proven benefits for both hepatic and musculoskeletal health (129). A sustained reduction of 7%–10% in body weight has been shown to significantly reduce hepatic steatosis and can even lead to histological remission of NASH in many cases. Even modest weight loss (~5%) can lower liver fat content and improve aminotransferase levels (129). However, weight reduction must be achieved gradually and in a muscle-preserving manner. Rapid or excessive caloric restriction without adequate exercise may accelerate muscle wasting and bone loss, as lean mass tends to decline alongside fat mass during dieting, and reduced mechanical loading may compromise BMD (130). Clinical trials in MASLD have demonstrated that hypocaloric diets improve metabolic parameters and liver fat content but may result in significant lean mass loss when protein intake or resistance training is insufficient (130). Consequently, a moderate daily caloric deficit (~500 kcal/day), combined with adequate protein intake (≥1.0–1.2 g/kg/day in older adults) and resistance exercise, is recommended to preserve muscle and bone during weight loss. This approach is strongly endorsed by professional liver societies (e.g. EASL, AASLD) and supported by high-level evidence (130). Future research is increasingly focused on long-term adherence strategies and incorporating body composition tracking into weight management protocols for MASLD patients, particularly those at risk for sarcopenia (131).

#### 5.1.2 Diet quality

Beyond caloric balance, dietary composition plays a critical role in managing MASLD-associated osteosarcopenia (43, 132, 133). The Mediterranean diet—rich in fruits, vegetables, whole grains, legumes, lean proteins (especially fish and poultry), and healthy fats (such as olive oil and nuts)—has demonstrated efficacy in reducing hepatic fat, enhancing insulin sensitivity, and lowering systemic inflammation in MASLD (129). This dietary pattern is also abundant in bone-supportive nutrients, including calcium, magnesium, vitamin K, and polyphenols (134). While low-carbohydrate or low-fructose diets may offer hepatic benefits, caution is warranted to avoid inadequate protein intake. For older adults and patients with cirrhosis, guidelines recommend protein intakes of ≥1.0–1.2 g/kg/day and 1.2–1.5 g/kg/day, respectively, to counter hypercatabolism. Patients with sarcopenia should prioritize high-quality protein with each meal to stimulate muscle protein synthesis. In cirrhosis, small frequent meals and protein-rich evening snacks (often BCAA-enriched) are commonly used to prevent nocturnal muscle catabolism (134). Although direct evidence linking specific dietary patterns to fracture or sarcopenia outcomes in MASLD is limited, extrapolation from general geriatric populations is reassuring. For instance, a trial in postmenopausal women showed that adherence to a Mediterranean diet plus resistance exercise helped maintain BMD and enhanced muscle strength (134). Thus, emphasizing dietary quality—alongside caloric moderation—can provide substantial multi-organ benefits. Ongoing clinical trials comparing Mediterranean and low-fat diets in MASLD will offer further insight into their musculoskeletal effects.

#### 5.1.3 Exercise

Exercise remains one of the most effective interventions to counteract sarcopenia and has well-documented benefits for both bone and liver health (135). A combination of resistance training (to enhance muscle mass and strength) and weight-bearing aerobic exercise (to promote bone remodeling and metabolic improvement) is considered ideal (136). In MASLD, exercise has been shown to reduce hepatic steatosis and improve insulin sensitivity even in the absence of weight loss (137). Randomized trials have demonstrated that resistance training not only increases muscle strength and mass but also lowers liver fat content by improving glucose uptake in skeletal muscle (137). Concurrently, impact and resistance-based exercises provide mechanical loading necessary for bone maintenance or gain (137). For example, a six-month combined aerobic and resistance program has been associated with increased spine BMD and reduced biomarkers of fracture risk (137). In patients with compensated cirrhosis, supervised exercise regimens—such as walking and light resistance training—have been shown to improve physical function and muscle mass safely (137). The combination of exercise and nutritional supplementation (e.g. protein or BCAA-enriched formulas) offers additive benefits, outperforming either strategy alone in preserving lean mass (138). While strong evidence supports the role of physical activity in improving muscle function and liver outcomes, adherence remains a major barrier. Fatigue and comorbidities may limit patient participation (138). Tailored interventions—such as chair-based routines for frail individuals or aquatic therapy for those with joint pain—may enhance feasibility. Structured programs and behavioral support (e.g. telehealth, health coaching, or gamification) may further improve long-term adherence. Standard recommendations include at least 150 minutes per week of moderate-intensity activity and resistance exercises at least two to three times per week (138). Overall, exercise should be prioritized as a foundational therapy for MASLD-associated osteosarcopenia, with implementation strategies tailored to individual needs.

### 5.2 Nutritional supplements and support

Nutritional optimization forms a cornerstone in the management of osteosarcopenia in MASLD.

#### 5.2.1 Protein and amino acids

Adequate protein intake is critical, particularly in patients with sarcopenia (138). Many MASLD patients consume insufficient dietary protein, exacerbating muscle wasting. A high-protein diet—typically ≥1.2 g/kg/day—is recommended to support muscle protein synthesis and maintain bone health, as protein is essential for the formation of the bone collagen matrix (138). In cirrhotic patients, even higher intake (1.2–1.5 g/kg/day) with frequent meals is advised to counteract hypercatabolism (138). Supplementation with branched-chain amino acids (BCAAs)—especially leucine—has shown potential in preventing muscle loss (139). BCAAs activate mTOR signaling and promote muscle protein synthesis. Hiraoka et al. reported that nighttime oral BCAA supplementation combined with walking exercise significantly mitigated muscle loss over one year in patients with cirrhosis (140). β-hydroxy β-methylbutyrate (HMB), a leucine metabolite, has demonstrated improvements in muscle mass and strength in elderly sarcopenic individuals, particularly when combined with resistance training, although evidence in liver disease remains limited. These nutritional strategies are biologically plausible, low-risk, and supported by a growing body of evidence. Although data in MASLD are limited, their use in MASLD patients with or at risk of sarcopenia appears reasonable as an adjunct to overall protein repletion (139).

#### 5.2.2 Vitamin D and calcium

Correction of vitamin D deficiency is essential in individuals with low BMD or osteoporosis, and MASLD patients often exhibit low levels due to hepatic dysfunction, altered bile acid metabolism, and vitamin D sequestration in adipose tissue (141). The therapeutic target is a serum 25(OH)D concentration >30 ng/mL (139). Supplementation with 800–2000 IU/day of vitamin D and calcium intake of 1000–1200 mg/day (via diet or supplements) is recommended in standard osteoporosis care. In addition to improving calcium absorption and bone mineralization, vitamin D may modestly improve muscle function, especially in those with deficiency. Several trials in elderly populations have shown reduced fall risk following high-dose vitamin D supplementation (142, 143). One study in patients with chronic liver disease demonstrated that correcting vitamin D deficiency improved muscle mass and chair-stand performance, likely through inflammation reduction and improved calcium handling in muscle (139).

#### 5.2.3 Other micronutrients

Vitamin K2 (menaquinone) plays a role in carboxylating osteocalcin, a protein critical for bone mineralization. While not yet part of routine care, small studies suggest K2 may reduce bone loss and fracture risk (144). Gut dysbiosis associated with MASLD could impair K2 production, potentially limiting its availability (145, 146). Combined supplementation with vitamin D and K2 is an area of ongoing research.

Vitamin E (800 IU/day) is used in non-diabetic NASH for its anti-inflammatory properties. Although its direct effects on bone are unclear, reducing oxidative stress may support musculoskeletal health. However, long-term use of high-dose vitamin E may carry risks, such as increased prostate cancer risk reported in one trial (144). Selenium, zinc, and omega-3 fatty acids are also being explored for their roles in muscle function and inflammation, though specific guidelines for osteosarcopenia are lacking (147).

### 5.3 Osteoporosis medications

For MASLD patients who meet diagnostic criteria for osteoporosis or have sustained a fragility fracture, standard osteoporosis therapies should be implemented, with appropriate attention to liver-related considerations (148).

#### 5.3.1 Bisphosphonates

Alendronate and risedronate (oral), as well as zoledronic acid (intravenous), are first-line antiresorptive agents (149). These medications inhibit osteoclast activity, reduce bone resorption, and lower fracture risk by approximately 50% at the spine and 20–30% at the hip (116). In patients with chronic liver disease, most evidence comes from studies in primary biliary cholangitis (PBC), where alendronate significantly increased BMD at the spine and hip after one year of treatment compared to no therapy (116). Although fracture outcomes were not powered in these small trials, BMD gains were consistently observed (150). Additional small studies suggest bisphosphonates can stabilize or improve BMD in patients with cholestatic or viral cirrhosis (149).

By extension, patients with MASLD-related osteoporosis are expected to derive similar benefits from bisphosphonates, with no evidence suggesting reduced efficacy in this context (151, 152). Some practical considerations are worth noting: oral BPs can cause esophageal irritation, which raises concern in patients with esophageal varices—IV zoledronate may be preferred in such cases (153). Moreover, adequate calcium and vitamin D status should be ensured prior to initiating bisphosphonates, as MASLD patients are frequently deficient (149). Observational studies in cirrhosis suggest that patients treated with bisphosphonates have fewer fractures than untreated patients, although confounding may exist (55, 154). While no dedicated trials exist for bisphosphonates in NASH cirrhosis, treating MASLD-related osteoporosis according to general guidelines is currently appropriate (150). There is also speculation that bisphosphonates might modulate liver disease via effects on bone-derived signaling molecules, but this remains unproven and they are presently regarded as bone-specific in action (149).

#### 5.3.2 Denosumab

Denosumab, a monoclonal antibody against RANKL, is a potent antiresorptive administered subcutaneously every six months (149). It is as effective as bisphosphonates in increasing BMD and reducing spine and hip fractures. In patients with MASLD or cirrhosis, denosumab has several advantages: it causes no upper gastrointestinal side effects, does not rely on hepatic metabolism, and can be used safely in renal impairment (153). Small case series in liver disease (e.g., PBC) report significant BMD improvements with denosumab. For MASLD patients with esophageal varices or other contraindications to oral BPs, denosumab provides a convenient and effective alternative (153). However, calcium and vitamin D levels must be optimized before administration, as hypocalcemia can occur. There is theoretical interest in RANKL inhibition for its potential anti-inflammatory effects in MASLD-related liver fibrosis, but at present, denosumab is used purely for skeletal indications (116). Importantly, therapy discontinuation without transition to another antiresorptive may result in rebound bone loss, so continuity of care is critical.

#### 5.3.3 Anabolic agents

In patients with severe osteoporosis—such as those with very low BMD or prior vertebral fractures—anabolic therapies like teriparatide (PTH 1-34) or romosozumab (sclerostin antibody) may be indicated (153). Teriparatide, administered daily for up to two years, substantially increases bone density and reduces vertebral fracture risk by ~65% (149). It may be particularly beneficial in osteosarcopenic patients, where rapid bone formation is needed. Moreover, PTH may also enhance muscle function through IGF-1 upregulation, providing dual benefits in frail MASLD patients. Although data in cirrhosis are limited, teriparatide has been used without significant adverse events, with appropriate calcium monitoring (116).

Romosozumab offers dual action—stimulating bone formation and inhibiting resorption—with monthly injections over a 12-month period, resulting in large BMD gains and reduced fracture risk (150). However, it carries a black box warning for cardiovascular risk. Given that MASLD patients often have metabolic syndrome and increased cardiovascular risk, romosozumab should be used cautiously and avoided in patients with recent myocardial infarction or stroke. While anabolic agents do not directly treat sarcopenia, by preventing fractures and preserving mobility, they contribute indirectly to maintaining muscle function. These agents should be followed by antiresorptives to sustain BMD gains.

Osteoporosis in MASLD should be managed according to established osteoporosis treatment guidelines (149). Bisphosphonates and denosumab are considered first-line therapies, with the choice influenced by comorbid conditions and route of administration (116). While the presence of liver disease may guide practical decisions—favoring IV or SC administration in select cases—it should not delay or preclude treatment (150). These medications have robust evidence supporting their role in fracture prevention. Even in advanced liver disease, they are generally safe, except in rare cases such as decompensated cirrhosis with severe coagulopathy or malabsorption, where additional caution is warranted (155).

### 5.4 Sarcopenia-specific drugs

As of 2025, no pharmacologic agents have been officially approved for sarcopenia treatment (156). However, several promising drug classes are under investigation, with relevance to MASLD patients who often suffer from overlapping metabolic, hepatic, musculoskeletal, and endocrine dysfunction (157).

#### 5.4.1 Myostatin/ActRIIB inhibitors

Myostatin is a key negative regulator of muscle growth. Inhibiting the myostatin–activin type II receptor axis (e.g., bimagrumab) has shown increased lean mass in trials for muscle-wasting disorders (158). Yet, functional benefits have been modest, and side effects such as excessive fat loss have been observed, especially in trials for obesity and diabetes (157). In liver disease, myostatin expression is upregulated—partly due to hyperammonemia via NF-κB activation—contributing to muscle atrophy (157). Animal studies suggest that blocking this pathway can mitigate ammonia-induced sarcopenia. While no human trials have yet tested these inhibitors in cirrhosis or MASLD, they represent a mechanistically compelling target for future intervention (116).

#### 5.4.2 Selective androgen receptor modulators and hormonal therapies

SARMs are synthetic ligands that selectively stimulate androgen receptors in muscle and bone while sparing other tissues, minimizing androgenic side effects (158). Agents such as enobosarm have demonstrated lean mass gain and slight functional improvement in cancer cachexia and metabolic disorders (156). Unlike high-dose testosterone, SARMs are less hepatotoxic and may be suitable for MASLD patients with sarcopenia (158). For male patients with documented hypogonadism, testosterone replacement has shown benefits in muscle mass, bone density, and even reduced hepatic decompensation in cirrhotic populations (156). However, treatment requires careful monitoring for prostate health and erythrocytosis, and is contraindicated in active hepatocellular carcinoma. In women, low-dose estrogen or selective estrogen receptor modulators (SERMs) may benefit bone and potentially muscle through anti-inflammatory mechanisms (157). Observational studies suggest postmenopausal hormone therapy may slow fibrosis progression in MASLD, though data remain limited and risks such as thrombosis must be considered (116).

### 5.5 GH/IGF-1 axis augmentation

Both MASLD and cirrhosis are associated with reduced levels of GH and IGF-1, which play key roles in maintaining muscle and metabolic homeostasis (158). Augmenting this axis has shown therapeutic promise, though clinical applications remain limited (159).

Ghrelin mimetics such as ibutamoren (MK-677), a GH secretagogue, have demonstrated increased IGF-1 levels and lean mass in older adults (160). However, these benefits are accompanied by side effects including appetite stimulation and transient insulin resistance—potentially problematic in MASLD patients with existing metabolic dysregulation (156).

Direct IGF-1 therapy has been explored in small studies involving cirrhotic patients, with improvements in nitrogen balance observed (158). Nonetheless, IGF-1 carries risks such as hypoglycemia and fluid retention, limiting its broad use.

A more targeted approach involves GH replacement in GH-deficient individuals with MASLD (159). A small trial showed that GH therapy improved hepatic steatosis and fibrosis markers, highlighting the contribution of GH deficiency to MASLD progression (161). However, in individuals with normal GH levels, therapy may induce insulin resistance, restricting its generalizability (156).

Emerging strategies under investigation include local IGF-1 gene delivery to muscle and activation of the mTOR pathway, which may bypass systemic side effects (158). Though still experimental, these interventions offer mechanistic rationale for future treatment of sarcopenia, particularly in cirrhosis where IGF-1 is severely suppressed.

### 5.6 Gene and cell therapies

Although still experimental, gene-based approaches are being explored to address the root causes of muscle and bone wasting. For instance, delivery of the follistatin gene—which binds and inhibits myostatin—has led to significant muscle hypertrophy in animal models, essentially serving as long-term myostatin blockade. Similarly, gene therapy strategies that upregulate osteoprotegerin (OPG), a decoy receptor for RANKL, may reduce bone resorption (159). While far from clinical application, such strategies offer a conceptual framework for targeting osteosarcopenia at the genetic level (158).

Mesenchymal stem cell (MSC) therapy represents another promising avenue. MSCs have been studied in cirrhosis for their anti-fibrotic effects, but their potential to support muscle regeneration and enhance bone formation—via paracrine effects or direct differentiation—remains of interest. Though clinical evidence in MASLD-related osteosarcopenia is lacking, the multi-lineage potential of MSCs underscores a novel regenerative approach.

### 5.7 Gut microbiome modulation

As part of the gut–liver–bone axis, modulation of the gut microbiome through probiotics, prebiotics, or fecal microbiota transplantation (FMT) is gaining traction (162). A healthy microbiome can enhance production of short-chain fatty acids (SCFAs) like butyrate (anti-inflammatory and muscle-sparing), increase vitamin K2 synthesis, and reduce intestinal permeability and endotoxemia (163).

Small randomized trials in cirrhotic patients have shown that probiotics can reduce blood ammonia, improve muscle mass indices, and enhance handgrip strength—likely by reducing inflammation and ammonia burden (164). In MASLD, specific probiotics have reduced hepatic steatosis and ALT levels, and may also attenuate osteosarcopenia by lowering systemic inflammation (162). Prebiotics, by increasing butyrate levels, have been shown in animal studies to raise IGF-1 and stimulate bone formation. Though FMT remains experimental, it has demonstrated some metabolic benefits, and whether it can reverse muscle or bone deterioration is an area of active investigation. Overall, microbiome-targeted interventions may offer a holistic means of modulating liver, muscle, and bone health simultaneously.

### 5.8 Anti-inflammatory and anti-fibrotic agents

Several pharmacologic agents targeting hepatic inflammation and fibrosis are currently in clinical trials. These include obeticholic acid (a farnesoid X receptor [FXR] agonist), selonsertib (an apoptosis signal-regulating kinase 1 [ASK1] inhibitor), and tocilizumab (an IL-6 receptor antagonist). While their primary aim is to improve liver histology, secondary benefits on muscle and bone are possible via reduced systemic inflammation (164).

However, potential off-target effects must be considered. For example, FXR agonists may impair bone formation by inhibiting osteoblast activity, and phase III trials of obeticholic acid have raised concerns regarding increased cholesterol and adverse skeletal outcomes (165). IL-6 blockade could be bone-protective—given IL-6’s role in osteoclast activation—but may blunt muscle hypertrophy signaling. Thus, the net musculoskeletal impact remains uncertain (164).

### 5.9 Future directions

Given the multifactorial nature of osteosarcopenia in MASLD, combination therapy is increasingly recognized as necessary. Rather than relying on a single modality, concurrent interventions targeting distinct pathophysiologic pathways may synergistically enhance outcomes. A patient-centric regimen might integrate lifestyle modification (including dietary and physical activity interventions), nutritional supplementation, pharmacologic therapy for osteoporosis, and a MASLD-specific agent (165).

For instance, in an older patient with NASH and osteosarcopenia, a comprehensive strategy could involve a Mediterranean diet with adequate protein intake, vitamin D and calcium supplementation, resistance training combined with aerobic exercise, antiresorptive therapy such as alendronate or denosumab for osteoporosis, and semaglutide to address hepatic steatosis and obesity (164). This integrative approach simultaneously targets liver pathology, bone loss, and muscle decline. Indeed, the concept of therapeutic “bundles” has gained traction, with growing expert consensus supporting multimodal interventions over monotherapy. While individual therapies—such as branched-chain amino acids (BCAA), resistance exercise, or vitamin D—have limited standalone efficacy, their combination has demonstrated greater functional improvement in muscle strength and mass (166).

Nonetheless, critical evidence gaps remain. It is still unclear whether treating osteoporosis in MASLD patients translates to hepatic benefits such as reduced encephalopathy, or whether increasing muscle mass can attenuate fibrosis progression. While some components (e.g., weight loss, structured exercise) have robust evidence for improving MASLD outcomes, others—such as myostatin inhibitors or fecal microbiota transplantation—remain experimental (163). Future prospective trials are urgently needed to determine whether osteosarcopenia-targeted interventions can reduce adverse outcomes such as falls, fractures, or mortality in MASLD (163). Demonstrating such benefits would further validate osteosarcopenia as not merely a comorbidity, but a modifiable contributor to disease trajectory (162).

## 6 Conclusion

Osteosarcopenia in MASLD reflects a convergence of age-related musculoskeletal decline and metabolic liver injury, significantly compounding disease burden. Current evidence robustly supports several key points: insulin resistance, chronic inflammation, and other shared mechanisms underpin the co-occurrence of MASLD, sarcopenia, and osteoporosis; epidemiologic studies consistently show greater muscle and bone loss in individuals with MASLD; and the presence of osteosarcopenia is associated with adverse outcomes including frailty, fractures, and increased mortality. These findings are well-established across the literature.

However, important uncertainties persist. The causal pathways remain incompletely delineated—for example, the extent to which gut dysbiosis contributes to osteosarcopenia is still unclear. More critically, the optimal strategies to prevent or reverse osteosarcopenia in MASLD have yet to be defined. While promising avenues such as structured exercise programs and emerging pharmacotherapies were discussed, many interventions remain at an exploratory or preclinical stage.

Osteosarcopenia in MASLD ultimately exemplifies a multisystem disorder that demands a multidisciplinary response. Early recognition and comprehensive management—incorporating lifestyle modification, nutritional support, and appropriate pharmacologic treatment—may offer a path to improving both lifespan and health span. Future research should focus on identifying effective interventions and rigorously testing whether improving musculoskeletal health translates into fewer liver-related complications and improved survival. By shifting osteosarcopenia from a risk marker to a modifiable therapeutic target, we can meaningfully advance care for MASLD patients.
